# Development of High-Yielding Upland Cotton Genotypes with Reduced Regrowth after Defoliation Using a Combination of Molecular and Conventional Approaches

**DOI:** 10.3390/genes14112081

**Published:** 2023-11-15

**Authors:** Salman Naveed, Johnson Toyinbo, Hrishikesh Ingole, Prasanna Valavanur Shekar, Michael Jones, B. Todd Campbell, Sachin Rustgi

**Affiliations:** 1 Department of Plant and Environmental Sciences, Clemson University Pee Dee Research and Education Center, Florence, SC 29506, USA; snaveed@clemson.edu (S.N.); jtoyinb@clemson.edu (J.T.); hingole@clemson.edu (H.I.); pshekar@clemson.edu (P.V.S.); majones@clemson.edu (M.J.); 2 USDA-ARS Southern Regional Research Center, New Orleans, LA 70124, USA; 3 USDA-ARS Coastal Plains Soil, Water, and Plant Research Center, Florence, SC 29501, USA; todd.campbell@usda.gov

**Keywords:** upland cotton, regrowth after defoliation, floral clustering, fiber yield, fiber quality

## Abstract

Cotton is an economically important crop. However, the yield gain in cotton has stagnated over the years, probably due to its narrow genetic base. The introgression of beneficial variations through conventional and molecular approaches has helped broaden its genetic base to some extent. The growth habit of cotton is one of the crucial factors that determine crop maturation time, yield, and management. This study used 44 diverse upland cotton genotypes to develop high-yielding cotton germplasm with reduced regrowth after defoliation and early maturity by altering its growth habit from perennial to somewhat annual. We selected eight top-scoring genotypes based on the gene expression analysis of five floral induction and meristem identity genes (*FT*, *SOC1*, *LFY*, *FUL*, and *AP1*) and used them to make a total of 587 genetic crosses in 30 different combinations of these genotypes. High-performance progeny lines were selected based on the phenotypic data on plant height, flower and boll numbers per plant, boll opening date, floral clustering, and regrowth after defoliation as surrogates of annual growth habit, collected over four years (2019 to 2022). Of the selected lines, 8×5-B3, 8×5-B4, 9×5-C1, 8×9-E2, 8×9-E3, and 39×5-H1 showed early maturity, and 20×37-K1, 20×37-K2, and 20×37-D1 showed clustered flowering, reduced regrowth, high quality of fiber, and high lint yield. In 2022, 15 advanced lines (F_8_/F_7_) from seven cross combinations were selected and sent for an increase to a Costa Rica winter nursery to be used in advanced testing and for release as germplasm lines. In addition to these breeding lines, we developed molecular resources to breed for reduced regrowth after defoliation and improved yield by converting eight expression-trait-associated SNP markers we identified earlier into a user-friendly allele-specific PCR-based assay and tested them on eight parental genotypes and an F_2_ population.

## 1. Introduction

Cotton (*Gossypium* spp.) is one of the major sources of natural fiber and vegetable oil globally [1,2]. Despite advances in plant breeding and management practices, the cotton yield gain has stagnated. One reason for the stagnated yield is the narrow genetic base [1,3,4]. As a result of this narrow genetic base, breeding progress has slowed, which could represent an impediment to enhancing pest and disease resistance, improving fiber quality, and sustaining high yields in cotton cultivars [1,5,6,7,8,9]. One of the important factors in the success of the cotton industry is the development of high-yielding cultivars exhibiting enhanced pest and disease resistance, early maturity, high quality of fiber, and low management costs [1,10].

Genetic diversity is an important factor that may contribute to early crop maturity, fiber yield, and quality. The success of a breeding program is highly dependent on the diversity of the gene pool [11]. The intensive use of a few genotypes in a breeding program can lead to a narrow genetic base [12]. Earlier studies conducted on 260 commercial upland cotton cultivars, released between 1970 and 1990, to determine the coefficient of parentage and pedigrees showed a narrow genetic base [3,6,13,14]. Further investigations using molecular markers also confirmed the low genetic diversity in cultivated cotton germplasm [12,15,16,17,18,19,20].

The introgression of the beneficial variations in the existing cotton germplasms using conventional and molecular plant-breeding approaches helps widen the cotton genetic base. For example, the interspecific hybridization between *Gossypium barbadense* with fine-quality long staple fibers but low yield and *Gossypium hirsutum* with high yield and low fiber quality led to the transmission of the desired fiber quality traits from *G. barbadense* to *G. hirsutum* [21,22]. On the other hand, *G. hirsutum* contributed favorable alleles for other fiber-related characteristics such as fiber length, strength, and micronaire [21,23]. These studies suggested that the allelic combinations of different genes contributing to desirable traits could be achieved via interspecific hybridization followed by selection [24]. Additionally, Soomro et al. [25] reported that intraspecific hybrids in *G. hirsutum* and intraspecific hybrids in *G. barbadense* showed 33.7% and 28.3% heterosis, respectively.

Genotypes exhibiting traits that fall beyond the phenotypic range of the parental genotypes, known as transgressive segregation, are commonly occurring phenomena affecting different quantitative traits in interspecific/intraspecific hybrids. The interspecific cotton populations that segregate for different phenotypic traits, such as plant height (short or tall), flowering time (early or late), boll size (small or large), pre/post-harvest regrowth (less or more), maturity (early or late), and fiber fineness (coarse to fine), were developed in the past [26]. These extreme phenotypes are due to the dominant and recessive alleles inherited from the parental genotypes, and their different combinations in the filial generations give rise to these segregants with extreme phenotypes. However, it is not easy to understand the genetic basis of these segregants completely. Introgression breeding is a long-term process. However, using introgression breeding, cotton breeders and geneticists have in the past century developed different lines showing many desirable traits such as Acala-type fiber quality and Delta-type fiber yield with resistance to Fusarium wilt [27]. One major challenge of using traditional breeding techniques is the unintentional transfer of undesirable genes to the next generation, making the pyramiding of desirable genes lengthy.

Gene pyramiding is defined as stacking desirable genes into a single genetic background using conventional and molecular breeding techniques. Several approaches have been used for gene stacking. One such approach is identifying trait-associated DNA markers and their use in marker-assisted selection. An example of this approach is the identification of quantitative trait loci (QTL) for growth habit in cotton [28]. Gene pyramiding is one of the most popular approaches to crop improvement by stacking genes for resistance to different biotic and abiotic stresses [28,29,30,31,32,33,34].

Plant architecture is an important factor that determines growth habit, maturity, crop management, and productivity. Meristems determine the plant architecture, which can be determinate (consumed during the flower production) or indeterminate (supporting reiterative vegetative growth). The meristem identity is determined by members of the PEBP (phosphatidylethanolamine-binding protein) gene family, such as *CETS* (*CENTRORADIALIS/TERMINAL FLOWER 1/SELF PRUNING*) and *FLOWERING LOCUS T* (*FT*). One of the members of this gene family plays an important role in promoting the determinate growth habit in plants by serving as a key component of the florigen activation complex (FAC), which activates the expression of downstream meristem identity genes *SUPPRESSOR OF OVEREXPRESSION OF CONSTANS 1* (*SOC1*), *LEAFY* (*LFY*), *APETALA 1* (*AP1*), and *FRUITFUL* (*FUL*). On the other hand, reduced expression of the cotton *FT*, *SOC1*, and *FUL* genes supports vegetative growth, delayed flowering, and bushy architecture in cotton [35]. The development of cotton cultivars with somewhat annual growth habit having fewer vegetative branches and reduced regrowth after defoliation, which are desirable traits in cotton, has led to compact plants with reduced vegetative and more reproductive growth with improved yield under optimal growth conditions, which facilitate mechanical picking [35,36,37].

Early maturity is one of the key breeding objectives in cotton breeding programs. In cotton, early maturity is a complex trait that includes different indicators such as growth habit, first fruiting branch node (FFBN), the height of the first fruiting branch (HFFBN), flowering time, bud period, boll opening date, and boll maturity. Different phenotypic traits have been considered to evaluate early maturity in cotton, but FFBN was the most reliable indicator of early maturity [38,39]. The value of the FFBN has a direct relationship with plant height and earliness of the onset of squaring, flowering, and boll opening [40]. Likewise, the timing of FFBN appearance is a key indicator of early maturity in cotton [41], as early-maturing cotton shows lower FFBN and HFFBN values [42]. The indicators of early maturity are typically quantitative traits determined by QTL and the environment [43,44]. Simultaneous selection for these agriculturally important traits using a conventional plant breeding approach is challenging. In the recent past, the rapid use of molecular markers, next-generation sequencing (NGS), and QTL mapping have helped researchers to investigate the genetic architecture of these quantitative traits and utilize this knowledge to improve crop plants.

In this study, we used an upland cotton mini-core collection of 44 genotypes to develop cotton genotypes with reduced regrowth after defoliation and improved fiber yield by altering the growth habit of plants from perennial to somewhat annual. With these alterations, we expect changes in the plant architecture, height, flowering time, boll number, internode length, and pre/post-harvest regrowth. To achieve this objective, we collected tissues at three developmental stages, stage 1 (S1)—10 days after sowing (DASs), stage 2 (S2)—30 DASs, and stage 3 (S3)—45 DASs, respectively, from cotyledonary leaves, immature square, and mature square, for three consecutive years from 2017 to 2019 to study expression patterns of five floral induction and meristem identity genes, *FT*, *SOC1*, *LFY*, *AP1*, and *FUL* and identify high-expression alleles of these genes [45]. We hypothesized that the high-expression alleles of these signal integrators and meristem identity genes would result in plants with annual growth habit exhibiting reduced to no regrowth after defoliation and enhanced yield, as the assimilates would be channeled towards fiber yield over storage in buds for regrowth. To identify high-expression alleles of selected genes, we developed an arbitrary expression matrix, where any genotype showing an expression level more than the population mean at a developmental stage in a study year was given a point. The genotypes were sorted from high to low expression levels [45] to make genetic crosses with an aim to stack the high-expression alleles of floral induction and meristem identity genes in a single genetic background. The specific objectives of the study were to (i) stack the complementing high-expression alleles of five floral induction and meristem identity genes by genetic crossing of selected genotypes; (ii) evaluate selected lines for surrogate traits for annual growth habit; (iii) develop molecular markers for reliable screening of genotypes with reduced regrowth after defoliation.

## 2. Materials and Methods

### 2.1. Plant Material

At the Pee Dee Research and Education Center (PDREC), we had access to 44 of the 53 upland cotton mini-core collection genotypes [46]. For the remaining nine genotypes, insufficient seed was available for propagation. Hence, in this study, 44 upland cotton genotypes were included. These genotypes were cultivated at PDREC in the same field (34°18′39″ N 79°44′40″ W) consecutively for three years, from 2017 to 2019. This mini-core collection represents over 92% diversity of a larger collection of the upland cotton genotypes released in the USA in the past 100 years [45]. A list of 44 genotypes used in this study is presented in Table S1.

### 2.2. Crop Husbandry

As mentioned earlier, the upland cotton genotypes of the mini-core collection were cultivated consecutively for three years from 2017–2019 in PDREC field # 7 (34°18′39″ N 79°44′40″ W). Genetic crosses between selected lines were made in 2018 and 2019, and in subsequent years, the progeny of crosses made from selected cotton genotypes with high-expression alleles of five floral induction and meristem identity genes were evaluated in field # 23 in 2020 (34°17′30″ N 79°44′34″ W), field # 45 in 2021 (34°18′04″ N 79°44′05″ W), and field # 31 in 2022 (34°16′54″ N 79°44′35″ W). All fields are located at the Clemson University PDREC and were managed similarly over the years. In the years 2020–2022, the plants were at F_2_/F_3_ to F_6_/F_7_ stage. Every year, the seeds of selected lines were advanced at the Cotton Winter Nursery (CWN), Liberia, Costa Rica.

Before sowing at PDREC or dispatching seed for CWN, the cotton seeds were ginned and delinted in the USDA-ARS delinting facility at PDREC, and the delinted seeds were treated with fungicides (composition: 10% Allegiance metalaxyl, Bayer Crop Sciences, Research Triangle, NC, USA, 3.3% Trilex trifloxystrobin, Bayer Crop Sciences, USA, 0.66% Vortex ipconazole, Bayer Crop Sciences, USA, and 3.3% EverGol penflufen, Bayer Crop Sciences, USA). The seeds were sown in 40-foot two-row plots with a 1-foot plant-to-plant distance. On average, 200 seeds per genotype were mechanically sown (100 seeds/row). The seeds were planted 2–3 cm deep, the fields were periodically sprayed with insecticides and herbicides, and defoliants were applied before physiological maturity to facilitate harvesting. Generally, the defoliants were applied onto the plants in mid to late September each season. The active ingredients of the defoliants included tribufos (a cell wall disrupter), diuron (a photosynthesis inhibitor), thidiazuron (an auxin inhibitor), and ethephon (a plant growth regulator), procured from Bayer Crop Sciences, USA and applied at the rate of 0.26, 0.84, 0.42, and 2.24 kg/ha, respectively. The harvesting was performed by manually picking bolls to ensure seed purity for generation advancement and mechanically to obtain the plot yield.

### 2.3. Genetic Crossing and Advancement of Generation

Eight upland cotton genotypes selected for high-expression level of the five floral induction and meristem identity genes (*FT*, *SOC1*, *LFY*, *AP1*, and *FUL*) were selected for making genetic crosses. These genotypes were selected from a screen of the 44 upland cotton mini-core collection lines for the high-expression alleles of the selected cotton genes at three developmental stages from 2017–2019 using qRT-PCR (for details, see ref. [45]). Collectively, we recorded data for 45 gene expression traits, i.e., five genes and three developmental stages, for three consecutive years (5 × 3 × 3). The gene expression data for these genes were normalized to the cotton housekeeping gene *ACT4-2*. An arbitrary point system was developed to facilitate the selection process. In this system, each individual genotype was given a point for an expression trait when it was found to express a gene at a developmental stage more than the population mean of that gene’s expression in a given year. In this way, an individual genotype could receive a maximum of 45 points (5 genes × 3 developmental stages × 3 years) [45]. The genetic crosses between selected cotton genotypes with complementary expression patterns of the selected genes were made reciprocally in the field in the 2018 and 2019 growing seasons following the commonly used procedure.

The bolls from crossed plants were manually harvested, ginned, and delinted, and the generations were advanced utilizing the CWN and the cotton research fields at PDREC. The phenotypic data on various traits such as plant height, flower and boll numbers per plant, boll opening date, floral clustering, and regrowth after defoliation were recorded in alternative generations, and selections were made. We used the single-seed descent method for the advancement of generations. A timeline of the generation advancement is given in Figure S1.

In 2022, fifteen advanced breeding lines (F_6_ and F_5_ generations) were cultivated in a triplicated randomized complete block design, and the phenotypic data collected from the trial were analyzed using Microsoft Excel and SAS packages.

### 2.4. Conversion of SNP Markers to User-Friendly PCR-Based Assay

The sequences of the eight selected markers showing associations with different expression traits [45] were retrieved from the CottonGen database (https://www.cottongen.org, accessed on 11 March 2021) to design allele-specific PCR-based assays. The full-length gene sequences provided sufficient flanking sequences to develop the allele-specific primer pairs. In these primer pairs, one primer’s (forward/reverse) 3′-end was tagged at the two alternative SNP alleles. Additionally, to improve the primer specificity, we introduced a non-template-specific nucleotide change at (n-2 location) (see Table S2). The primers were synthesized, and optimum annealing temperatures were determined using gradient PCR (Table S2) and validated on eight genotypes selected for genetic crossing and a GSA 74 (17) × TAMCOT SP-23 (39) F_2_ population. To test the specificity of the allele-specific primers, DNA was isolated from a month-old true tender leaf using the Mag-Bind^®^ Plant DNA DS Kit (Omega Bio-Tek Inc., Norcross, GA, USA) following the manufacturer’s instructions.

## 3. Results and Discussion

### 3.1. Intraspecific Hybridization

**Figure 1:**
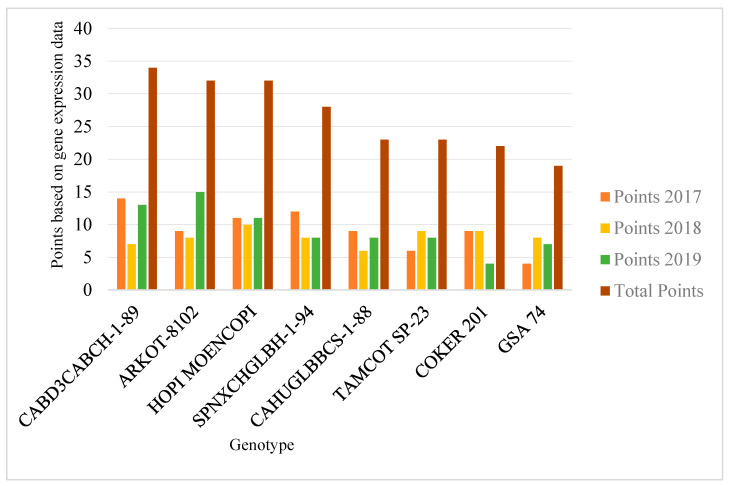
Eight cotton genotypes scored the most data points in the gene expression study of five floral induction and meristem identity (*FT*, *LFY*, *SOC1*, *AP1*, and *FUL*) genes at three developmental stages (cotyledonary leaf, first square, and subsequent square) studied for three consecutive years (2017, 2018, and 2019) (for further details, see ref. [45] and Section 2).

Based on the gene expression data for two years (2017 and 2018), we initially selected five genotypes, ARKOT-8102 (5), CABD3CABCH-1-89 (8), GSA-74 (17), SPNXCHGLBH-1-94 (37), and TAMCOT SP-23 (39) for making reciprocal genetic crosses to stack the desirable alleles in a single genetic background (Figure 1). A range of 14–22 genetic crosses was made per genotype combination (Table S3), and the resulting F_1_ cotton bolls were manually harvested from the field. The seeds were delinted and sent to Costa Rica to advance a generation. Further, in 2019, based on the gene expression data for three years (2017 to 2019), three more genotypes, CAHUGLBBCS-1-88 (9), COKER-201 (10), and HOPI MOENCOPI (20), were selected to make reciprocal genetic crosses (Figure 1) (for details, see ref. [45]). About 17 to 19 crosses per genotype combination were made. Collectively, 587 reciprocal genetic crosses of 30 genotype combinations were made in 2018 and 2019 (Table S3).

### 3.2. Generation Advancement and Phenotypic Evaluation

#### 3.2.1. F_2_ and F_3_ Generations

In the 2019 growing season, the genetic material was sown in two phases. In phase 1 (21 May 2019), 44 upland cotton genotypes of the mini-core collection were sown, and in phase 2 (23 May 2019), F_1_ and F_2_ populations were sown (Tables S3 and S4). Seeds were received in time for propagation in 2019 for five of twenty genetic crosses that were prioritized for the advancement of the generation in CWN. These crosses were prioritized due to the high expression scores carried by the crossed genotypes in our expression analysis. To sum up, a total of five F_2_ populations, 15 F_1_ populations (not sent to Costa Rica), and 44 upland cotton genotypes of the mini-core collection were planted in field # 7 (34°18′39″ N 79°44′40″ W) at the PDREC, where we had plots in the previous years (2017 and 2018). The primary objective behind sowing at the same site was to avoid edaphological differences. Precisely, 93 to 107 F_2_ plants per cross combination [CABD3CABCH-1-89 (8) × ARKOT-8102 (5); GSA 74 (17) × TAMCOT SP-23 (39); SPNXCHGLBH-1-94 (37) × TAMCOT SP-23 (39); CABD3CABCH-1-89 (8) × TAMCOT SP-23 (39); and GSA 74 (17) × ARKOT-8102 (5)] germinated (Table S4). Similarly, 37 to 95 F_1_s from 15 cross combinations germinated (Table S5). Bolls were manually harvested from F_2_ plants of each population and seeds from ten selected plants per cross combination (5 crosses per combination) were sent to CWN for generation advancement (Table S6).

**Figure 2:**
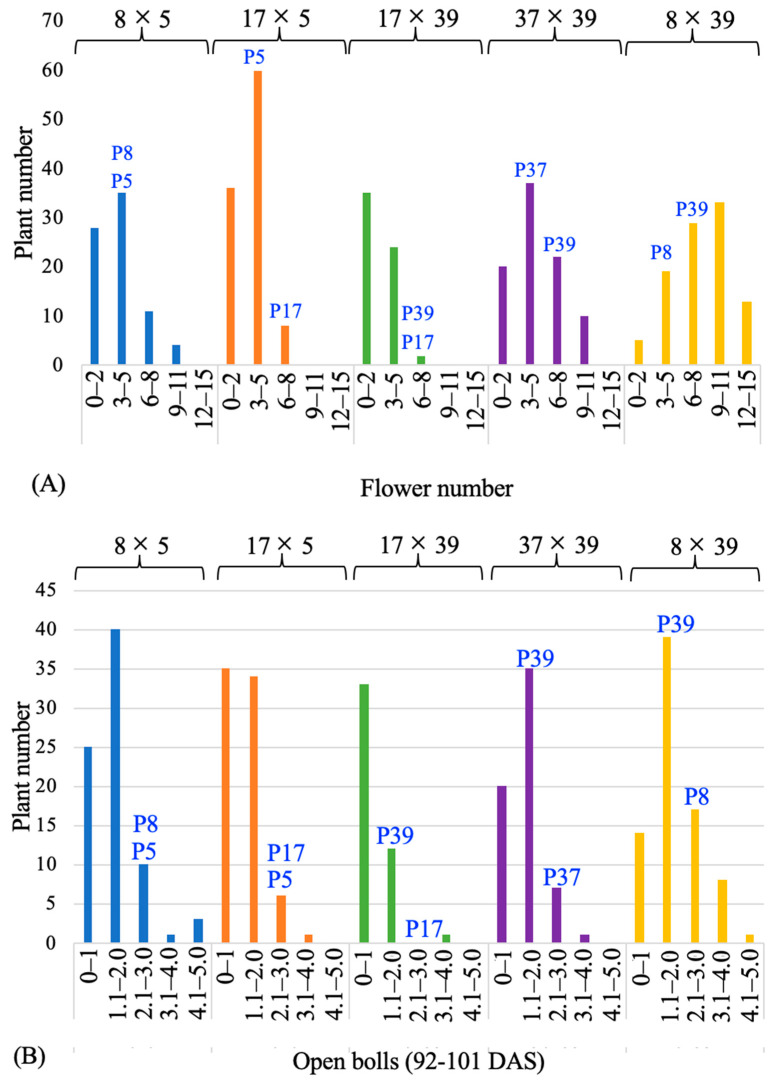
Plots showing the distribution of the number of flowers formed between 15 July 2019 and 22 July 2019 (53 to 60 days after sowing) per plant (**A**) and boll opening date (**B**) in five F_2_ cotton populations. The IDs of parental genotypes are labelled on the bars reflective of the phenotype class they belong to; for the genotype names, see Table S1. DAS = days after sowing.

Phenotypic data were collected on surrogate traits for annual/determinate growth habit, such as plant height, flower number per plant, and first boll opening date. The individuals of the F_2_ population showed transgressive segregation for these phenotypic traits. Precisely, four of the five F_2_ populations showed transgressive segregation for flower number, as F_2_ lines produced more flowers than the parental genotypes 53 to 60 days after sowing. Similar transgressive segregation was observed for the number of open bolls, recorded on 92 to 101 days after sowing (Figure 2). The boll opening date faithfully reflects flowering time and hence was recorded in this study to find early-flowering genotypes. Likewise, plant height is positively correlated with boll number and negatively correlated with earliness in cotton, somewhat reflective of annual/determinate growth habit; hence, it was recorded for each population. In general, in all five populations, the plants showed more compact stature than the parental genotypes. This is in line with our hypothesis that the strong expression alleles of the five floral induction and meristem identity genes stacked together will promote an annual/determinate growth habit with a more compact plant form.

Furthermore, we isolated the DNA from one of the F_2_ populations, GSA 74 (17) × TAMCOT SP-23 (39), to test whether any of the expression-trait-associated markers identified in our previous work showed an association with the surrogate phenotypic traits recorded on the F_2_ population [45]. For this purpose, we converted SNPs to user-friendly PCR-based assays. We genotyped 91 F_2_ lines of the GSA 74 (17) × TAMCOT SP-23 (39) population with four SNP markers, i09222Gh, i00443Gh, i13158Gh, and i13851Gh (Table S7). Unfortunately, a variable number of plants produced no data, resulting in missing data points.

#### 3.2.2. F_3_ and F_4_ Generations

Thirty different populations were developed by crossing eight selected upland cotton genotypes in various combinations (Table S3). Out of these populations, four F_3_ populations and ten F_2_ populations were sown in the field at PDREC in the 2020 growing season. The wet and cold weather conditions and the field’s location in a low-lying area (leading to waterlogging in some plots) resulted in poor plant turnout in some populations. Specifically, three F_2_ populations, which were crosses between ARKOT-8102 (5) × CABD3CABCH-1-89 (8), TAMCOT SP-23 (39) × CABD3CABCH-1-89 (8), and ARKOT 8102 (5) × TAMCOT SP-23 (39) either showed no germination or poor survival after germination. The F_3_ population derived from the reciprocal genetic cross between CABD3CABCH-1-89 (8) × TAMCOT SP-23 (39) also exhibited poor germination. However, due to the confounding effect of the environmental conditions, it is difficult to conclude whether any of the observed effects on germination were genetic.

The parental genotypes and the surviving individuals of F_2_/F_3_ populations were selfed in the field conditions. Phenotypic data on the plant height, total boll number per plant, first boll opening date, and regrowth after defoliant application were recorded. Simple linear regression of plant height and total boll number and plant height and percentage of open bolls was performed, and the analysis showed a positive correlation between plant height and total boll number (almost all populations) and a negative/no correlation between plant height and percentage of open bolls (Figure S3). Interestingly, the correlation between plant height and total boll number was much more robust in populations involving ARKOT-8102 (5) and/or TAMCOT SP-23 (39), hinting towards the genetic nature of this correlation. The plants selected for propagation in Costa Rica are marked on the regression/correlation plots to make it easy to understand the bases of plant selection. The criteria for plant selection are further elaborated in Tables S8 and S9.

#### 3.2.3. F_4_ and F_5_ Generations

In 2020, 25 F_3_ lines belonging to four genetic crosses [SPNXCHGLBH-1-94 (37) × TAMCOT SP-23 (39), GSA 74 (17) × ARKOT 8102 (5), CABD3CABCH-1-89 (8) × TAMCOT SP-23 (39), and CABD3CABCH-1-89 (8) × ARKOT 8102 (5)] and 28 F_2_ lines belonging to seven genetic crosses [CABD3CABCH-1-89 (8) × CAHUGLBBCS-1-88 (9), CAHUGLBBCS-1-88 (9) × ARKOT 8102 (5), HOPI MOENCOPI (20) × ARKOT 8102 (5), TAMCOT SP-23 (39) × ARKOT 8102 (5), SPNXCHGLBH-1-94 (37) × CABD3CABCH-1-89 (8), CABD3CABCH-1-89 (8) × SPNXCHGLBH-1-94 (37), and HOPI MOENCOPI (20) × SPNXCHGLBH-1-94 (37)] were sent to Costa Rica to advance a generation. Unfortunately, a poor germination rate was observed across all genotypes; precisely, only 8 out of 28 F_2_ lines germinated, and 7 out of them yielded seeds. Similarly, 4 out of 25 F_3_ lines germinated, and only 2 produced seeds. We could not find out the precise reason for poor germination but attributed it to severe flooding caused by Hurricane Eta that struck Costa Rica (3–5 November 2020). Most of this material was re-sown at PDREC in 2021. In 2021, sowing took place on 11 June in the PDREC cotton research field (delayed as the seed from Costa Rica was received on 9 June 2021). Precisely, the seeds of the advanced generations, including F_3_/F_4_ (received from Costa Rica) and F_2_/F_3_ (seeds sent the previous year to Costa Rica but which did not germinate), were planted.

**Figure 3:**
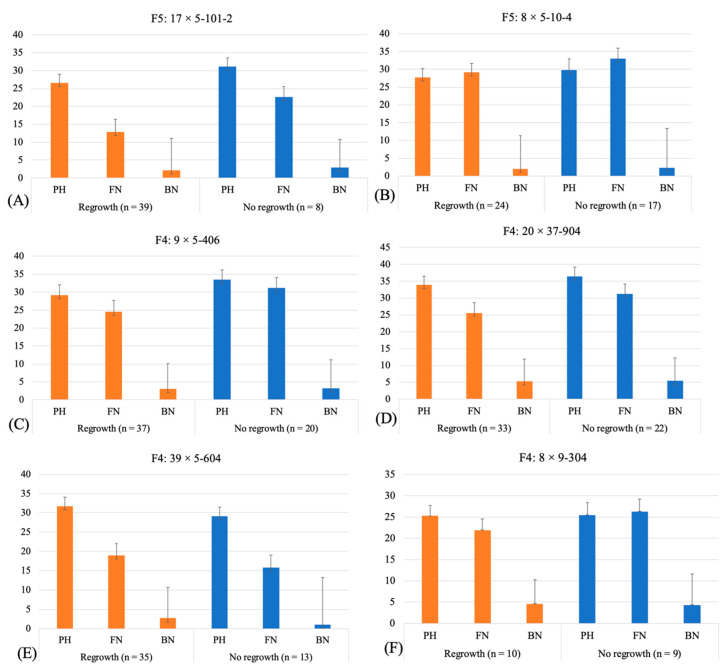
The bar charts show the averaged values for plant height, number of flowers per plant, and number of bolls per plant in the plants of 17 × 5-101-2 (**A**) and 8 × 5-10-4 (**B**) F_5_ populations, and 9 × 5-406 (**C**), 20 × 37-904 (**D**), 39 × 5-604 (**E**), and 8 × 9-304 (**F**) F_4_ populations showing regrowth and no regrowth. For genotype names, see Table S1. PH = average plant height (in inches); FN = average flower number; and BN = average boll number.

Phenotypic data on plant height, flower number (including candles and white, pink, and brown flowers) per plant, total bolls per plant, floral clustering, and regrowth after defoliation were collected from all field-grown plants. Out of 50 genotypes of different advanced generations (F_3_ and F_4_ seeds from Costa Rica and F_2_ and F_3_ seeds from PDREC), a variable number of plants established in the field for 39 genotypes, whereas none of the plants germinated or survived for 11 genotypes (Table S10). The data on regrowth after defoliation were recorded for all surviving plants of 39 genotypes on 27 October 2021. Individuals of all populations were split into two categories: plants showing regrowth or no regrowth after defoliation. Subsequently, to find any trend for plant height, flower number (total number of candles and flowers of different ages), and boll number in each class, we averaged the trait values and present the range in Figure 3. Contrary to expectation, this analysis suggested no correspondence between regrowth and plant height, flower number, or number of bolls. Out of 39 genotypes analyzed, all studied plants for 5 genotypes (four F_4_ and one F_3_ generation) showed regrowth, whereas, for the remaining 34 genotypes, a variable number of plants showed no regrowth, where the number of plants with no regrowth ranged from a single plant to several plants. Interestingly, the plants of F_5_ line 8 × 5-10-4 showed no regrowth in 41.5% of individuals and also exhibited floral clustering on the fruiting branches (Figure 3). Traits like plant height (PH) reflect on the plant’s stature and, to some extent, the growth habit (indeterminate/determinate) of its main stem and the lower indeterminate branches (nodes 2–4), whereas traits like boll number (BN) and, to some extent, flower number (FN; precisely the way we recorded it, where we counted different flower developmental stages individually) reflect earliness and determinacy, as the flowers represent determinate growth. Regrowth after defoliation is a trait that reflects on the change in the growth habit from perennial to annual, as regrowth after defoliation is a perennial trait. In sum, the genotypes in these populations indicated a transition in the plant growth habit and architecture. The seeds from the selected plants were sent to CWN to advance a generation (Tables S11 and S12).

#### 3.2.4. F_5_ and F_6_ Generations

The F_5_ seeds from the ten plants (selected based on the phenotypic data) of two cross combinations, GSA 74 (17) × ARKOT-8102 (5) and CABD3CABCH-1-89 (8) × ARKOT-8102 (5), and F_4_ seeds from a variable number of plants of six cross combinations, CAHUGLBBCS-1-88 (9) × ARKOT-8102 (5), HOPI MOENCOPI (20) × SPNXCHGLBH-1-94 (37), CABD3CABCH-1-89 (8) × CAHUGLBBCS-1-88 (9), TAMCOT SP-23 (39) × ARKOT-8102 (5), HOPI MOENCOPI (20) × ARKOT-8102 (5), and HOPI MOENCOPI (20) × SPNXCHGLBH-1-94 (37) were sent to CWN for increase and generation advancement (Tables S11 and S12). The F_6_ and F_5_ seeds were received on 15 April 2022. A replicated yield trial (with three biological replicates of each line), including a higher-yielding check, DP-493, and a higher-quality-fiber check, FM-958, in a randomized complete block design, was planted on 18 May 2022.

The phenotypic data were collected on plant height (inches), boll number per plant, number of open bolls, floral clustering, regrowth after defoliation, lint yield, and fiber quality traits. The phenotypic data were recorded from the replicated field trial between 23–31 August 2022 (Table 1). The regrowth data were collected between 7–10 October 2022, about two weeks after the defoliant application on 20 September 2022. The data were collected from thirty flagged plants from three two-row plots per genotype (45 plots for 15 selected genotypes). Later, bolls (excluding any green bolls) were manually picked from the same plants (between 28–29 September 2022) for pure seeds. The basic idea was to send the seed to Costa Rica to obtain pure (F_8_/F_9_) seeds for replicated trials in 2023 and determine the lint yield and fiber quality. The plots’ mechanical harvest (boll picking) was performed on 28 October 2022 to obtain the yield data.

**Figure 4:**
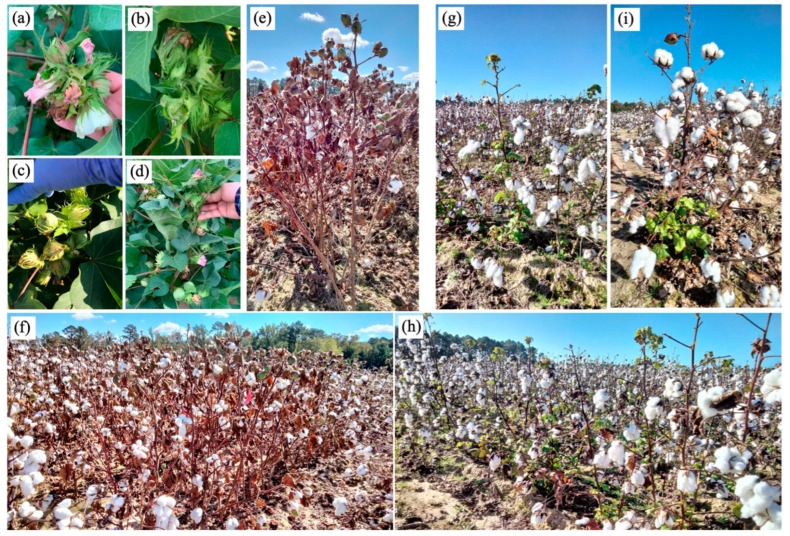
Cotton genotypes showing (**a**–**d**) clustered flowering [HOPI MOENCOPI (20) × SPNXCHGLBH-1-94 (37) cross], (**e**,**f**) reduced regrowth after defoliation [HOPI MOENCOPI (20) × SPNXCHGLBH-1-94 (37) cross], (**g**,**h**) terminal and basal regrowth (DP-493), and (**i**) basal regrowth (FM-958).

The data analysis suggested that in these selected advanced lines, we have lines appropriate for long growing seasons (such as the Carolinas) and short growing seasons (such as Texas). Genotypes such as CABD3CABCH-1-89 (8) × ARKOT-8102 (5)-B3, CABD3CABCH-1-89 (8) × ARKOT-8102 (5)-B4, CAHUGLBBCS-1-88 (9) × ARKOT-8102 (5)-C1, CABD3CABCH-1-89 (8) × CAHUGLBBCS-1-88 (9)-E2, CABD3CABCH-1-89 (8) × CAHUGLBBCS-1-88 (9)-E3, and TAMCOT SP-23 (39) × ARKOT-8102 (5)-H1 matured early (about 60% open bolls as early as September 1, 2022), dropped leaves without defoliant application, and exhibited compact stature. In contrast, genotypes HOPI MOENCOPI (20) × SPNXCHGLBH-1-94 (37)-K1, HOPI MOENCOPI (20) × SPNXCHGLBH-1-94 (37)-K2, and HOPI MOENCOPI (20) × SPNXCHGLBH-1-94 (37)-D1 exhibited clustered flowering, reduced regrowth after defoliant application, and produced a large number of bolls (Table 1; Figure 4). However, some of these later bolls did not mature in time for harvest during the manual picking between 28–29 September 2022, and during the recording of regrowth data between 7–10 October 2022, there were still green bolls on the plants (sprayed with defoliant on 20 September 2022). We hypothesized that the change in plant growth habit had reduced the internode length, resulting in floral clustering, and most of the buds turned into flowers, leading to some late-emerging bolls. However, as these plants set many bolls, the number of bolls produced offset the effect of not being able to harvest all bolls on yield, as most plants of the HOPI MOENCOPI (20) × SPNXCHGLBH-1-94 (37)-K2 family yielded significantly more than the high-yielding control, DP-493 (Table 2). Also, in case of a long growing season, the producers could delay harvest or plan multiple pickings, which may further enhance the yield. On the other hand, the short-statured early-maturing genotypes are suitable for areas with a shorter growing season as these mature early and could avoid drought and heat stress occurring later during the growing season. These early results suggest that the various combinations of the high-expression alleles of the floral induction and meristem identity genes (*FT*, *LFY*, *AP1*, *SOC1*, and *FUL*) lead to various outcomes, as the selected genotypes that we crossed carried high-expression alleles of different floral induction and meristem identity genes. Samples were collected from alternative generations of selected plants (grown at PDREC) for RNA (three developmental stages: cotyledonary leaves, immature square, and subsequent square) and DNA extractions. The objective of this ongoing effort is to track the expression pattern of the five floral induction and meristem identity genes and alleles of molecular markers associated with expression traits [45,47].

The fiber quality analysis of the selected genotypes showed some genotypes, such as HOPI MOENCOPI (20) × SPNXCHGLBH-1-94 (37)-D1, to carry high-quality-fiber relative to the high-quality-fiber variety FM-958 (Table 2). On the other hand, HOPI MOENCOPI (20) × SPNXCHGLBH-1-94 (37)-K2 family plants that exhibited many desirable traits, such as clustered flowering, reduced regrowth after defoliation, and high yield, exhibited desirable micronaire values but less desirable values for fiber length and strength (Table 2). We assumed this was because some of the bolls were not as mature as other bolls at harvest, resulting in the blending of mature and immature fibers, leading to reduced fiber-length and -strength values.

Subsequently, plants were selected for increase in Costa Rica based on the total number of bolls per plant, plant height (close to check varieties), floral clustering, earliness, and no-regrowth after defoliation. A total of 59 plants were selected to be ginned, delinted, and treated with fungicide. Additionally, 15 plants were selected and sent for increase to CWN (Table S13). These plants will be reevaluated at PDREC for two years (2023 and 2024) for the above-listed attributes in addition to fiber yields and quality, with an intent to release them as germplasm lines.

**Figure 5:**
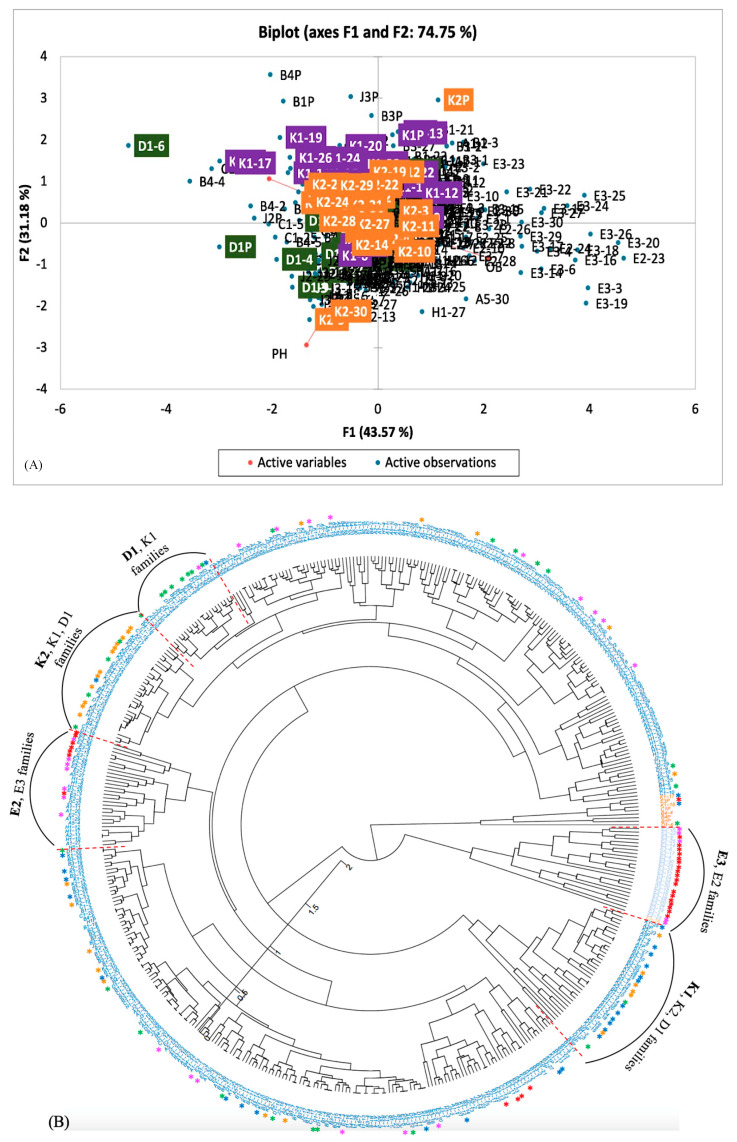
A principal component analysis (PCA) plot (**A**) and a neighbor-joining, unrooted, circular-dendrogram (**B**) showing the relationships among 15 cotton populations (a total of 450 lines; 15 plots × 3 replicates × 10 randomly selected plants per plot) derived from seven genetic crosses of seven selected upland cotton genotypes. The analysis was based on phenotypic data (plant height, boll number, and the number of open bolls). Genotype names are based on the family name followed by plant numbers 1–30 in the PCA plot, (**A**) and the dendrogram, (**B**). Notice the grouping of genotypes of a family with their siblings, as evident from following the font colors, e.g., clustering of K1, K2, and D1 family members [derived from the HOPI MOENCOPI (20) × SPNXCHGLBH-1-94 (37) cross]. However, the clustering of some individuals with members of other families can also be witnessed, which reflects the breakage of correlations between traits and could be an outcome of recombination. D1 = green “*”, E2 = pink “*”, E3 = red “*”, K1 = blue “*”, and K2 = orange “*”.

Furthermore, we used the phenotypic data (plant height, total number of bolls per plant, and the number of open bolls) recorded for ten randomly selected plants of 15 advanced cotton selections replicated thrice (a total of 450 plants) in the field to study the genetic relations among these families. These populations were derived from seven genetic cross combinations of seven selected upland cotton genotypes, HOPI MOENCOPI, SPNXCHGLBH-1-94, ARKOT-8102, TAMCOT SP-23, CABD3CABCH-1-89, CAHUGLBBCS-1-88, and GSA 74 with high-expression alleles of five floral induction and meristem identity genes [45]. As expected, using the phenotypic data, the related families (siblings) clustered together. However, the cross-clustering of genotypes with members of other families was also witnessed. This cross-clustering indicated breakage of correlations between traits, such as a positive correlation between plant height and total boll number per plant, probably due to recombination and stacking of high-expression alleles of five floral induction and meristem identity genes in different combinations (Figure 5).

### 3.3. Conversion of the Expression-Trait-Associated SNP Markers into User-Friendly PCR-Based Assays for Use in Cotton Breeding

**Figure 6:**
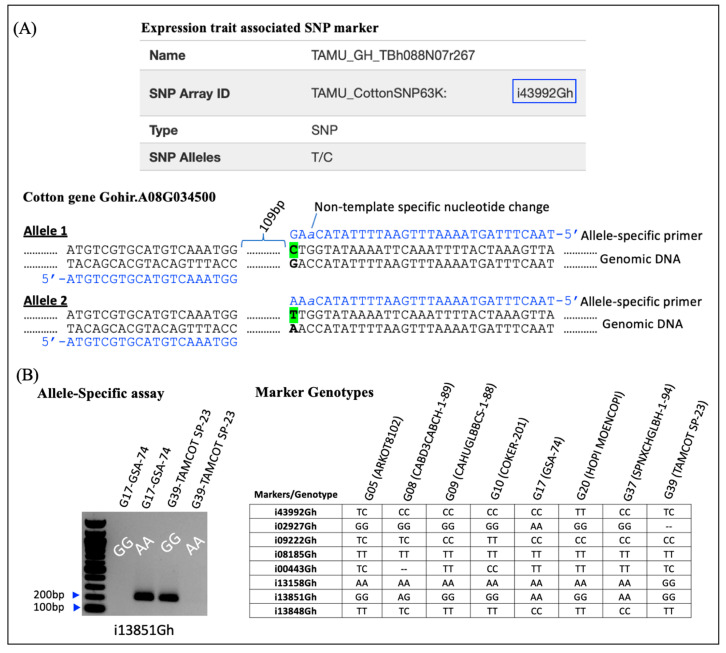
Diagram showing an expression-trait-associated SNP, the steps we followed to convert it into a PCR-based assay, and the steps forward to test it on the parental genotypes of the populations we developed to stack the expression traits. The marker information was retrieved from the CottonGen database and used to obtain the full-length gene sequence, in this case, *Gohir.A08G034500*. The two SNP alleles are colored fluorescent green. The allele-specific primers designed are tagged at their 3′-ends on the SNP, and as specified in the text, a non-template-specific nucleotide change (shown in a lower-case letter) is introduced at the n-2 location to enhance primer specificity. The common forward primer used with both allele-specific primers is shown (**A**). An example of the PCR-based assay developed for marker ‘i13851Gh with ‘G’ and ‘A’ specific primers on two parental genotypes is shown (**B**).

The sequences of the eight selected markers showing associations with different expression traits were retrieved from the CottonGen database to design allele-specific PCR-based assays that breeders could conveniently use to transfer and stack these traits in other relevant upland cotton breeding materials. We used the short SNP marker sequences (often about 100 nucleotides in length) to pull out the full-length gene sequences from the database. The full-length gene sequences provide sufficient flanking sequences to develop the allele-specific primer pairs (Table S3). In these primer pairs, one primer’s (forward/reverse) 3′-end was tagged at the two alternative SNP alleles (Figure 6A).

Additionally, to improve the primer specificity, we followed a strategy tested by Liu et al. [48] and introduced a non-template-specific nucleotide change at the n-2 location from the 3′-end of the oligonucleotide, where n is the SNP. The objective of introducing a non-template-specific (random) change at the n-2 location from the 3′-end is to destabilize the allele-specific primer and stop the production of non-specific products from the alternative SNP allele [49]. To test the specificity of the allele-specific primers, these were used with the genomic DNA of parental genotypes. Primers designed from the *18S rRNA* gene were used as a positive control in each experiment. Under optimized PCR conditions, agarose gel electrophoresis allowed the differentiation of the lines from the 17 × 39 F_2_ population, whether they were homozygous or heterozygous for that particular expression-trait-associated SNP marker [50]. All eight (i02927Gh, i43992Gh, i13158Gh, i09222Gh, i00443Gh, i08185Gh, i13848Gh, i13851Gh) expression-trait-associated SNP markers were tested on eight parental genotypes (Figure 6B) and four (i13851Gh, i09222Gh, i13158Gh, and i00443Gh) of these markers were used on one F_2_ population (17 × 39). Out of a total of 91 lines from the 17 × 39 F_2_ population, various numbers of plants did not produce any data, resulting in missing data. For example, SNP marker i09222Gh was tested on 41 lines (45.05%), i13851Gh on 35 lines (38.46%), and i13158Gh and i00443Gh markers on 29 lines (31.86%). As stated, a variable number of lines from these populations did not produce any results, which is likely to be due to the presence of additional SNPs stacked in crossed progeny, making primer binding difficult and leading to a failed product; this possibility needs further investigation [51,52,53].

## 4. Conclusions

Gene pyramiding is an important technique to stack desirable genes in a genotype. This technique has successfully been used in different crops, including tomato, wheat, and rice, to develop biotic- and abiotic-stress-resistant cultivars. In this study, we used a novel strategy to improve cotton yield and fiber quality by altering its growth habit from an indeterminate to a more determinate type. The diversity of the germplasm used here allowed the creation of new allelic combinations via intraspecific hybridization, which made it possible to break undesirable combinations (e.g., positive correlation between plant height and flower number) and stacking of desirable phenotypes in a single genetic background, such as high flower number, early maturity, floral clustering, reduced regrowth after defoliation, and compact plant architecture. As expected, different genotypic combinations led to different allelic combinations of the floral induction and meristem identity genes that resulted in various lines exhibiting different characteristics, which is evident from the early-maturing lines [CABD3CABCH-1-89 (8) × ARKOT-8102 (5)-B3, CABD3CABCH-1-89 (8) × ARKOT-8102 (5)-B4, CAHUGLBBCS-1-88 (9) × ARKOT-8102 (5)-C1, CABD3CABCH-1-89 (8) × CAHUGLBBCS-1-88 (9)-E2, CABD3CABCH-1-89 (8) × CAHUGLBBCS-1-88 (9)-E3, and TAMCOT SP-23 (39) × ARKOT-8102 (5)-H1], which could perform well in the short growing season as in Texas, and high-yielding lines with reduced regrowth after defoliation, such as HOPI MOENCOPI (20) × SPNXCHGLBH-1-94 (37)-K1, HOPI MOENCOPI (20) × SPNXCHGLBH-1-94 (37)-K2, and HOPI MOENCOPI (20) × SPNXCHGLBH-1-94 (37)-D1, suitable for a long growing season like in the Carolinas.

In 2023, we received the seeds of the most advanced lines, F_9_/F_8_, from Costa Rica and are evaluating them in a randomized complete block design for a second consecutive year. This year, we are testing the performance of these advanced lines with the parental genotypes initially used for genetic crossing and the high-yielding and high-fiber quality checks, DP-493 and FM-958, respectively. This year’s and a subsequent year’s phenotypic evaluation will allow us to register and release these advanced breeding lines as germplasms. We believe these breeding lines will serve as a resource for the plant-breeding community to develop cotton cultivars with optimal flowering time and stature, clustered flowering, enhanced fiber yield and quality, and reduced regrowth after defoliation.

## Figures and Tables

**Table 1:** Phenotypic data recorded for selected cotton genotypes sown in triplicated randomized complete block design. DP-493 and FM-958 were used as high-yielding and high-quality-fiber controls, respectively. For genotype names, see Table S1.

Line	Plant Height Range (In)	Plant Height Average (In)	Boll Number (Range)	Boll Number Average	Open Bolls (Range) *	Floral Clustering (%)	Regrowth (%)
17×5-A1	27.5–41.5	36.84	6–27	15.1	0–5	0	100
17×5-A2	29.5–46	40.64	2–24	13.57	0–4	0	100
17×5-A5	30.5–43	38.83	8–27	16.5	0–8	0	100
8×5-B1	27–38.5	33.53	3–38	17.12	0–1	0	90
8×5-B3	28–38	34.45	3–37	19.67	0–1	0	86.67
8×5-B4	34–43	39.48	9–73	25.97	0–3	0	100
9×5-C1	31.5–45	39.47	2–44	20.67	0–2	0	56.67
8×9-E2	32.5–47.5	38.12	7–47	18.87	0–14	0	86.67
8×9-E3	28.5–39	33.48	4–23	11.87	0–13	0	86.67
39×5-H1	31.5–45	39.93	4–23	14.4	0–5	0	76.67
20×5-J2	31.5–47.5	39.85	4–29	15.73	0–4	0	100
20×5-J3	33–48	41.88	5–25	15.2	0–1	0	76.67
20×37-D1	32–47	39.75	10–96	22.63	0	3.33	80
20×37-K1	28.5–38	35.37	8–68	31.13	0	53.33	16.67
20×37-K2	33–49.5	38.65	6–47	25.23	0–1	56.67	30
DP-493	33.5–42.5	38.17	9–44	22.13	0	0	100
FM-958	30–44	38.17	13–49	29.57	0–4	0	100

* Data recorded between 23–31 August 2022.

**Table 2:** Lint yield recorded on per plot basis (obtained from cotton picker) and fiber quality data for selected cotton genotypes in each plot (SD values calculated and presented next to each quality parameter). DP-493 and FM-958 were used as high-yielding and high-quality-fiber controls, respectively.

Line	Lint Yield/Plot (lb)	Micronaire	Length	Uniformity	Strength
17×5-A1-B1	11.10	4.84 ± 0.23	1.12 ± 0.05	81.10 ± 1.17	31.30 ± 1.78
17×5-A1-B2	13.30				
17×5-A1-B3	10.50				
**Mean**	11.63 ± 1.47				
17×5-A2-B1	16.90	4.84 ± 0.20	1.06 ± 0.09	81.03 ± 1.41	31.30 ± 1.22
17×5-A2-B2	16.70				
17×5-A2-B3	15.00				
**Mean**	16.20 ± 1.04				
17×5-A5-B1	13.90	4.57 ± 0.13	1.09 ± 0.04	79.27 ± 2.27	31.33 ± 1.06
17×5-A5-B2	11.10				
17×5-A5-B3	12.80				
**Mean**	12.60 ± 1.41				
8×5-B1-B1	16.40	4.86 ± 0.21	1.02 ± 0.01	82.23 ± 0.31	31.93 ± 1.81
8×5-B1-B2	9.30				
8×5-B1-B3	14.10				
**Mean**	13.27 ± 3.62				
8×5-B3-B1	15.80	4.76 ± 0.18	1.06 ± 0.06	82.53 ± 1.06	32.30 ± 1.69
8×5-B3-B2	12.00				
8×5-B3-B3	12.40				
**Mean**	13.40 ± 2.09				
8×5-B4-B1	15.40	4.68 ± 0.37	1.08 ± 0.05	82.70 ± 0.98	30.30 ± 0.35
8×5-B4-B2	14.40				
8×5-B4-B3	11.90				
**Mean**	13.90 ± 1.80				
9×5-C1-B1	17.10	5.15 ± 0.50	1.16 ± 0.06	81.50 ± 1.42	31.25 ± 1.23
9×5-C1-B1	17.10				
9×5-C1-B1	14.10				
**Mean**	16.10 ± 1.73				
20×37-D1-B1	17.90	4.11 ± 0.59	1.24 ± 0.08	83.84 ± 1.12	34.04 ± 1.86
20×37-D1-B2	19.20				
20×37-D1-B3	11.50				
**Mean**	16.20 ± 4.12				
8×9-E2-B1	11.70	3.27 ± 0.79	0.98 ± 0.02	79.80 ± 1.17	25.50 ± 2.03
8×9-E2-B2	11.90				
8×9-E2-B3	9.90				
**Mean**	11.17 ± 1.10				
8×9-E3-B1	13.00	4.04 ± 0.68	0.92 ± 0.07	79.50 ± 2.69	24.50 ± 2.55
8×9-E3-B2	9.00				
8×9-E3-B3	8.50				
**Mean**	10.17 ± 2.47				
39×5-H1-B1	14.60	5.26 ± 0.24	0.99 ± 02	80.93 ± 1.07	28.10 ± 1.60
39×5-H1-B2	16.00				
39×5-H1-B3	12.50				
**Mean**	14.37 ± 1.76				
20×5-J2-B1	13.50	4.57 ± 0.08	1.09 ± 0.05	79.73 ± 1.48	27.35 ± 1.21
20×5-J2-B2	19.50				
20×5-J2-B3	13.40				
**Mean**	15.47 ± 3.49				
20×5-J3-B1	15.20	4.51 ± 0.11	1.09 ± 0.02	81.60 ± 0.47	27.25 ± 0.96
20×5-J3-B2	14.70				
20×5-J3-B3	14.40				
**Mean**	14.77 ± 0.40				
20×37-K1-B1	19.60	5.06 ± 0.16	0.94 ± 0.01	82.30 ± 0.57	27.00 ± 1.01
20×37-K1-B2	18.00				
20×37-K1-B3	17.60				
**Mean**	18.40 ± 1.06				
20×37-K2-B1	22.10	3.90 ± 0.18	0.98 ± 0.01	82.17 ± 0.61	27.83 ± 0.78
20×37-K2-B2	21.20				
20×37-K2-B3	20.20				
**Mean**	21.17 ± 0.95				
DP-493-B1	17.70	4.65 ± 0.37	1.12 ± 0.07	83.08 ± 1.54	33.16 ± 2.21
DP-493-B2	17.00				
DP-493-B3	18.60				
**Mean**	17.77 ± 0.80				
FM-958-B1	18.20	5.04 ± 0.26	1.21 ± 0.04	84.96 ± 1.05	35.94 ± 1.17
FM-958-B1	20.90				
FM-958-B1	14.10				
**Mean**	17.73 ± 3.42				
**LSD_0.05_**	1.34				

## Data Availability

The data presented in this paper are available on request from the corresponding author.

## Supplementary Materials

The following supporting information can be downloaded at: https://www.mdpi.com/article/10.3390/genes14112081/s1, Figure S1: Timeline of the advancement of the breeding lines; Figure S2. The regression plots made using the phenotypic data collected in F_2/3_ populations; Table S1: List of upland cotton genotypes used in the present study; Table S2: List of allele-specific primers developed for the expression-trait-associated SNPs for use in PCR-based assays and *18S rRNA* used as positive control; Table S3: List of genetic crosses made among five selected upland cotton genotypes during 2019; Table S4: List of the F_2_ populations (received from Costa Rica) sown at the PDREC; Table S5: List of the F_1_ genotypes sown at the PDREC; Table S6: List of F_1_ lines sent for generation advancement in Costa Rica at the winter nursery 2019–2020; Table S7: Genotyping of the F_2_ population (17 × 39) with the allele-specific molecular markers; Table S8: List of F_2_ plants selected for propagation in Costa Rica in 2020; Table S9: List of F_3_ plants selected for propagation in Costa Rica in 2020; Table S10: List of the populations sown at PDREC research fields in 2021; Table S11: List of F_4_ plants selected for propagation in Costa Rica; Table S12: List of F_5_ plants selected for propagation in Costa Rica; Table S13: List of allele-specific primers developed for the expression-trait-associated SNPs for use in PCR-based assays and *18S rRNA* used as positive control.
