# Supplementary material for: Development of High-Yielding Upland Cotton Genotypes with Reduced Regrowth after Defoliation Using a Combination of Molecular and Conventional Approaches
Source: Genes (Basel). 2023 Nov 15;14(11):2081. doi: 10.3390/genes14112081 (PMC10671241; doi:10.3390/genes14112081)
Supplement: Supplementary file 1 [file genes-14-02081-s001.zip › genes-2667806-supplementary.pdf]

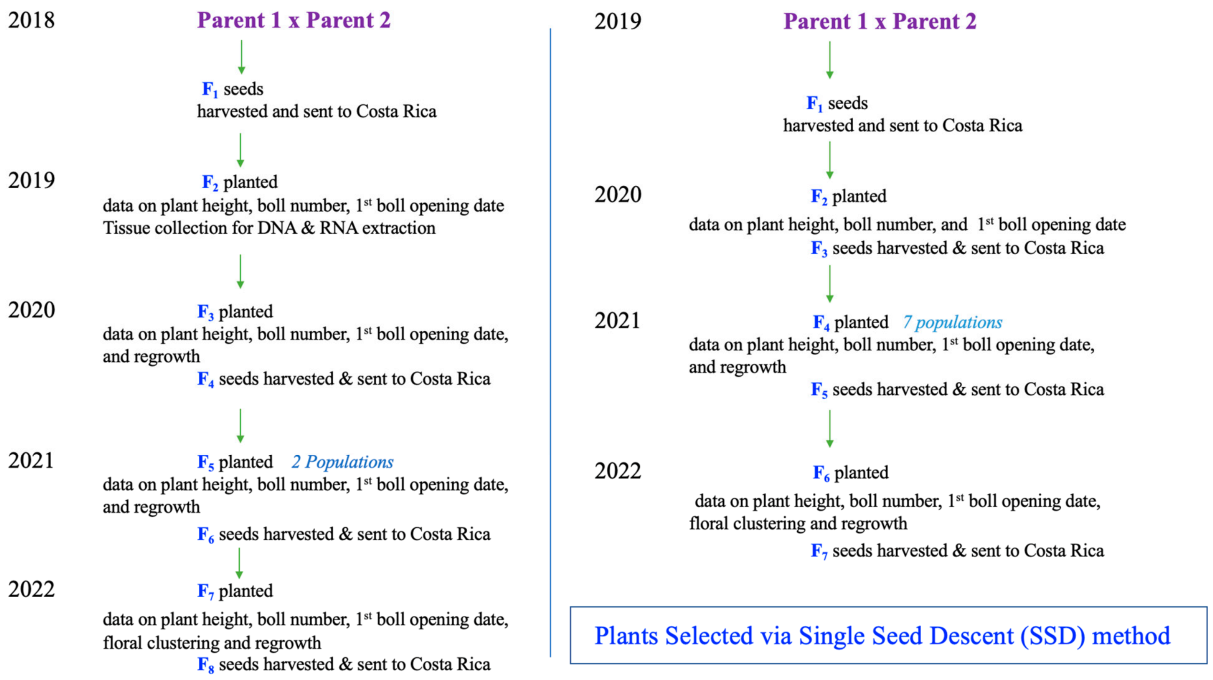

Figure S1. Timeline of the advancement of the breeding lines.

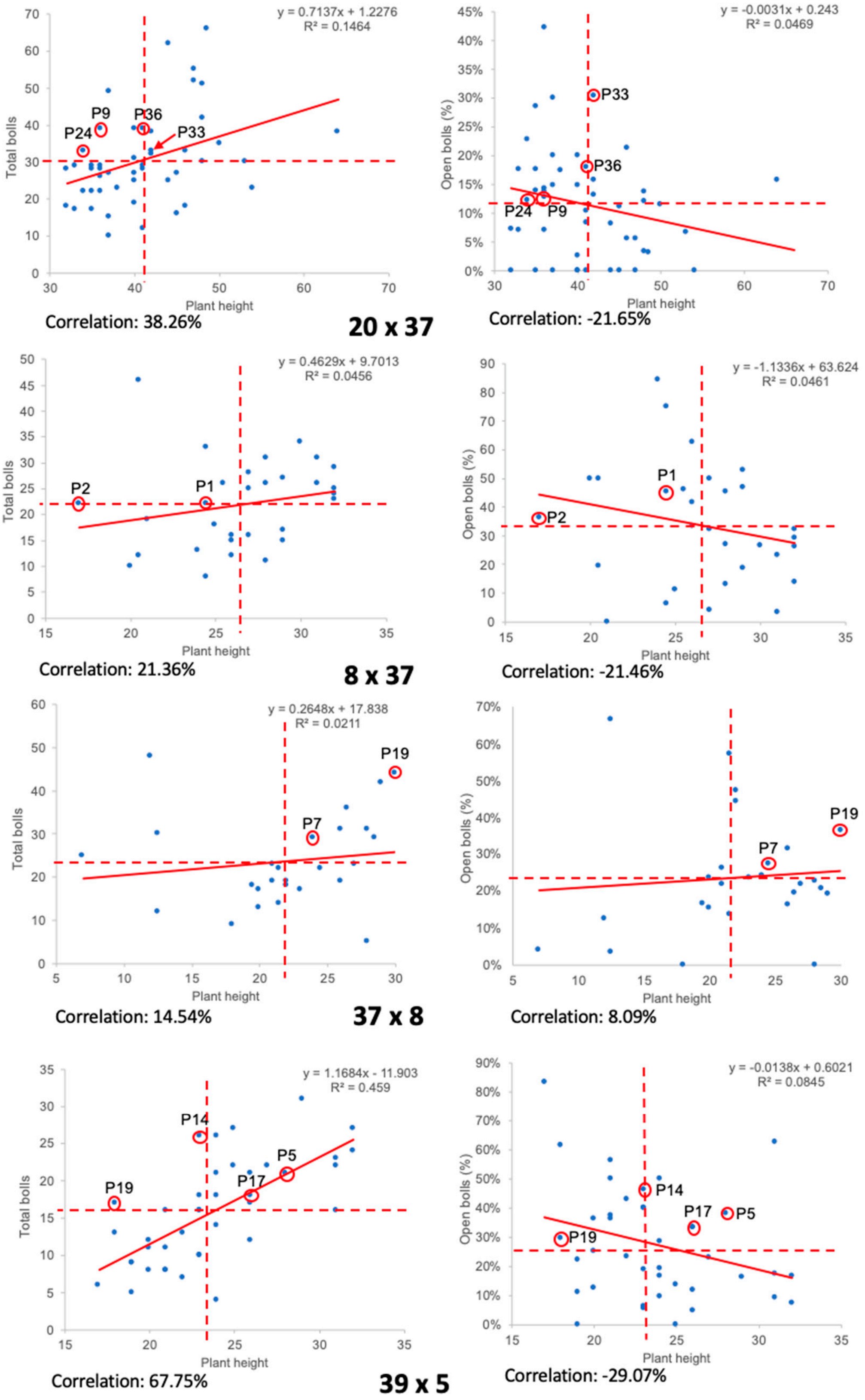

y = 0.7137x + 1.2276
R² = 0.1464
Total bolls
Plant height
P9
P24
P36
P33
Correlation: 38.26%
20 x 37
y = -0.0031x + 0.243
R² = 0.0469
Open bolls (%)
Plant height
P33
P36
P24
P9
Correlation: -21.65%
y = 0.4629x + 9.7013
R² = 0.0456
Total bolls
Plant height
P2
P1
Correlation: 21.36%
8 x 37
y = -1.1336x + 63.624
R² = 0.0461
Open bolls (%)
Plant height
P1
P2
Correlation: -21.46%
y = 0.2648x + 17.838
R² = 0.0211
Total bolls
Plant height
P19
P7
Correlation: 14.54%
37 x 8
Open bolls (%)
Plant height
P19
P7
Correlation: 8.09%
y = 1.1684x - 11.903
R² = 0.459
Total bolls
Plant height
P14
P5
P17
P19
Correlation: 67.75%
39 x 5
y = -0.0138x + 0.6021
R² = 0.0845
Open bolls (%)
Plant height
P14
P17
P5
P19
Correlation: -29.07%

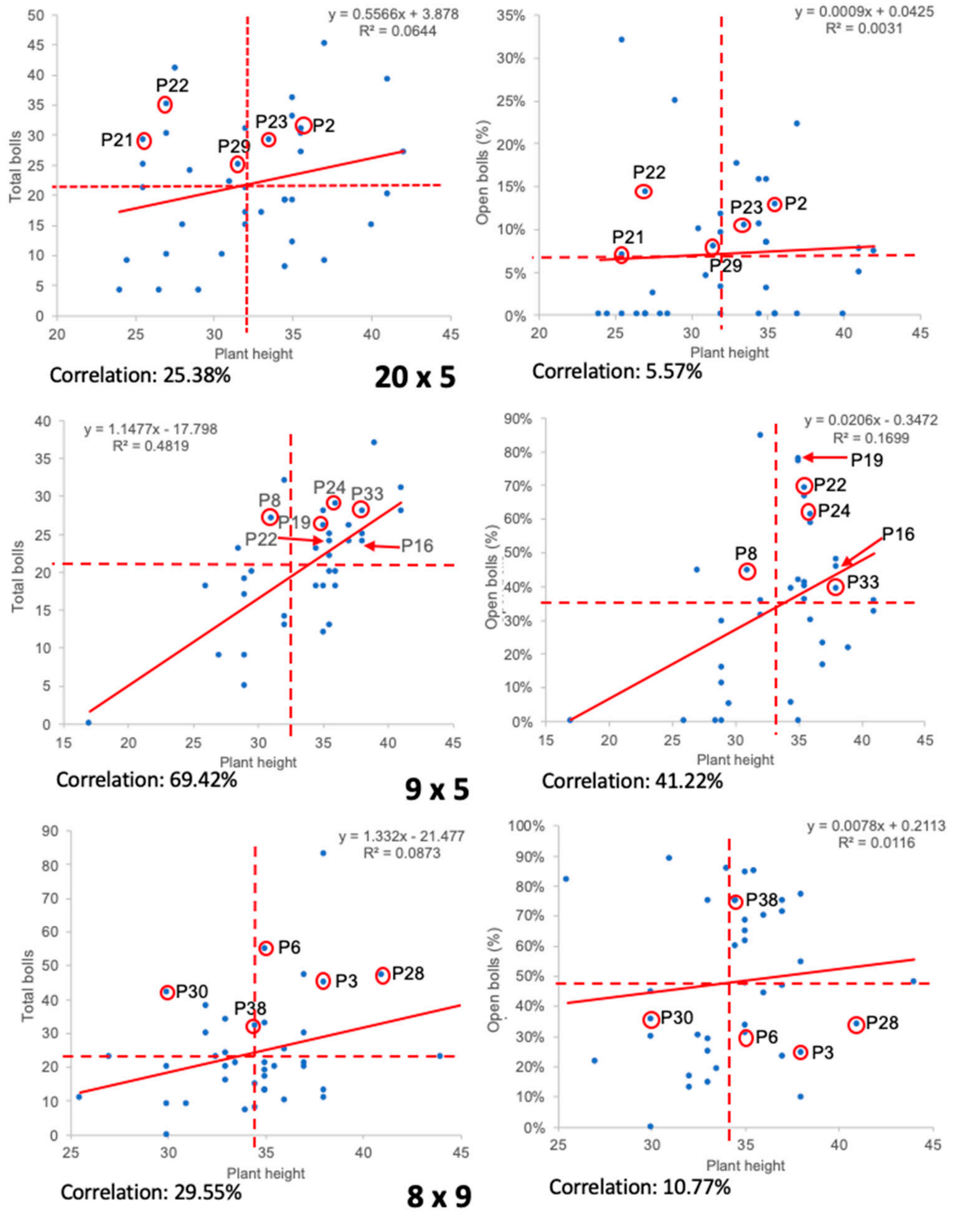

y = 0.5566x + 3.878
R² = 0.0644
Total bolls
Plant height
P22
P21
P29
P23
P2
Correlation: 25.38%
20 x 5
y = 0.0009x + 0.0425
R² = 0.0031
Open bolls (%)
Plant height
P22
P23
P2
P21
P29
Correlation: 5.57%
y = 1.1477x - 17.798
R² = 0.4819
Total bolls
Plant height
P8
P24
P33
P19
P22
P16
Correlation: 69.42%
9 x 5
y = 0.0206x - 0.3472
R² = 0.1699
Open bolls (%)
Plant height
P19
P22
P24
P16
P8
P33
Correlation: 41.22%
y = 1.332x - 21.477
R² = 0.0873
Total bolls
Plant height
P6
P3
P28
P30
P38
Correlation: 29.55%
8 x 9
y = 0.0078x + 0.2113
R² = 0.0116
Open bolls (%)
Plant height
P38
P30
P6
P28
P3
Correlation: 10.77%

(A)

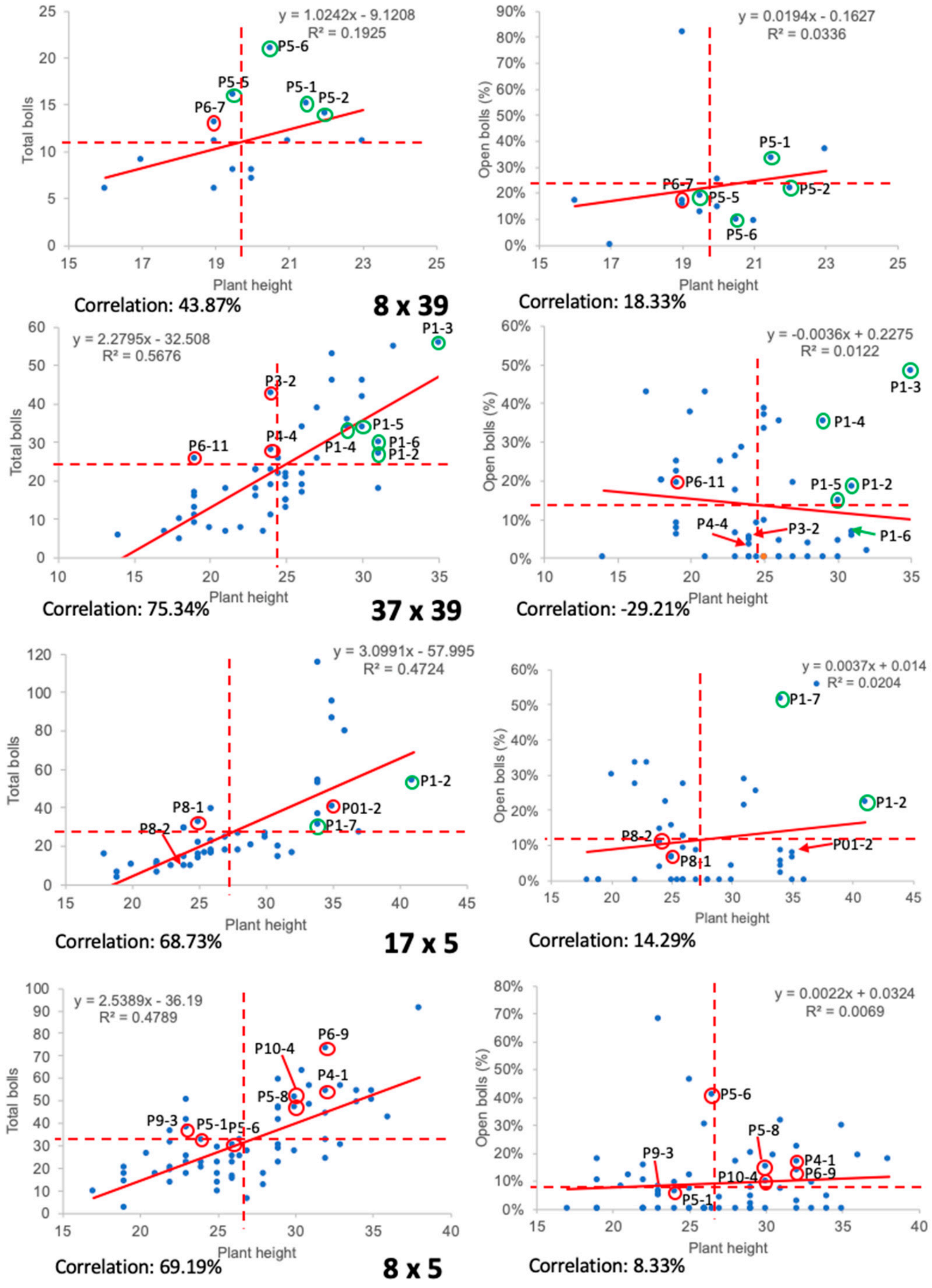

(B)

Figure S2. The regression plots made using the phenotypic data collected on plant height and total boll number (left) and plant height and percent open bolls (right) in seven F_2 populations, A and in four F_3 populations, B. Genotypes selected for propagation in Costa Rica are circled. The Pearson correlation values are provided below each plot, and the population means for plant height, total boll number, and percent open bolls are shown by dotted lines in each plot. The $F_{2/3}$ population is also labeled below the plot in bold letter. For genotype names, see Supplementary Table S1, S8, and S9.

Table S1. List of 44 Upland cotton minicore collection genotypes used in the present study.

Genotype ID	Genotype name
1	ACALA 111 ROGERS
2	ACALA 5
3	ALLEN 33
4	ARKANSAS 10
5	ARKOT 8102
6	BJAGL NECT
7	CA23
8	CABD3CABCH-1-89
9	CAHUGLBBCS-1-88
10	COKER 201
11	CS-8610
12	EARLISTAPLE 7
13	EMPIRE
14	EXPRESS 121
15	FJA
16	GREGG 35
17	GSA 74
18	H1330
19	HALF AND HALF
20	HOPI MOENCOPI
21	LZ.850082FN
22	LBBCDBOAKH-1-90
23	LOCKETT 88
24	M.U.8B UA 7-44
25	NC 88-95
26	NEW BOYKIN
27	PAYMASTER 101
28	PAYMASTER HS26
29	PD 0113
30	PD 781
31	PD 785
32	PD 93009
33	PD 93030
34	SEALAND #2
35	SEALAND #7 WHITE FLOWER
36	SOUTHLAND M1
37	SPNXCHGLBH-1-94
38	STATION MILLER
39	TAMCOT SP-23

40	TASHKENT 1
41	TIDEWATER 29
42	TOOLE
43	WANNAMAKER CLEVELAND
46	DELTAPINE 14

Table S2. List of allele-specific primers developed for the expression trait-associated SNPs for use in PCR-based assays and 18srRNA used as positive control. The two SNP alleles are shown in red, a non-template-specific nucleotide change is shown in blue color (lowercase). Common reverse or forward primers are shown in purple color.

**Associated SNP**	**Alleles**	**Primer name**	**Primer sequence (5′-3′)**
i02927Gh	A/G	i02927Gh _A_F	GTTTCTAGGCTAATACAACTTAcAA
		i02927Gh _G_F	GTTTCTAGGCTAATACAACTTAcAG
		i02927Gh _R	AGAGTGGTTCATTGTTGTTGTGA
i43992Gh	T/C	i43992Gh_F	ATGTCGTGCATGTCAAATGG
		i43992Gh_C_R	TAACTTTAGTAAAATTTGAATTTTATACaAG
		i43992Gh_T_R	TAACTTTAGTAAAATTTGAATTTTATACaAA
*i13158Gh	A/G	i13158Gh _A_F	CGGATTATTACAAGAAAGTTTGcCA
		i13158Gh _G_F	CGGATTATTACAAGAAAGTTTGcCG
		i13158Gh _R	CGGCGTGTGTTTTATCAGAGT
*i09222Gh	T/C	i09222Gh _C_F	GTCAATCATCTAACATCCTACTgAC
		i09222Gh _T_F	GTCAATCATCTAACATCCTACTgAT
		i09222Gh _R	TGTCATTGTCGTCGATCCCA
*i00443Gh	C/T	i00443Gh _T_F	CATTGTGGTGTTTCTGTcAT
		i00443Gh _C_F	CATTGTGGTGTTTCTGTcAC
		i00443Gh _R	TATCCTCCACATCCTCTGCC
i08185Gh	C/T	i08185Gh _F	AGGAAGAATGGAATGGGCAGA
		i08185Gh _C_R	AGGAATGGGAGGTGAGCgAG
		i08185Gh _T_R	AGGAATGGGAGGTGAGCgAA
i13848Gh	C/T	i13848Gh _C_F	GGTTCCGGAGCTGGCaC
		i13848Gh _T_F	GGTTCCGGAGCTGGCaT
		i13848Gh _R	GCCCTAAGTCAAAGCA
*i13851Gh	G/A	i13851Gh _F	TGACTCACTGTACATGGGCT
		i13851Gh _A_R	GTTGCCAAAGATTCTTCAATACTGAtTT
		i13851Gh _G_R	GTTGCCAAAGATTCTTCAATACTGAtTC
Positive control		18srRNA-F	TCTGCCCTATCAACTTTCGATGGTA
		18srRNA-R	AATTTGCGCGCCTGCTGCCTTCCTT

Note: Markers marked with an asterisk (*) are used for the validation on 91 lines of F_2 population (17x39) in this study.

Table S3. List of genetic crosses made among five selected Upland cotton genotypes (ARKOT-8102, CABD3CABCH-1-89, GSA-74, SPNXCHGLBH-1-94, and TAMCOT SP-23), during 2018 and three selected Upland cotton genotypes (CAHUGLBBCS-1-88, COKER-201, and HOPI MOENCOPI) during 2019.

Field ID	Female	Male	Crosses made
5	ARKOT-8102	CABD3CABCH-1-89	18
5	ARKOT-8102	GSA74	21
5	ARKOT-8102	SPNXCHGLBH-1-94	21
5	ARKOT-8102	TAMCOT SP-23	21
8	CABD3CABCH-1-89	ARKOT-8102	21
8	CABD3CABCH-1-89	GSA74	21
8	CABD3CABCH-1-89	SPNXCHGLBH-1-94	20
8	CABD3CABCH-1-89	TAMCOT SP-23	22
8	CABD3CABCH-1-89	CAHUGLBBCS-1-88	18
8	CABD3CABCH-1-89	COKER-201	18
8	CABD3CABCH-1-89	HOPI MOENCOPI	18
9	CAHUGLBBCS-1-88	ARKOT-8102	19
9	CAHUGLBBCS-1-88	COKER-201	18
9	CAHUGLBBCS-1-88	SPNXCHGLBH-1-94	17
17	GSA74	ARKOT-8102	20
17	GSA74	CABD3CABCH-1-89	20
17	GSA74	SPNXCHGLBH-1-94	22
17	GSA74	TAMCOT SP-23	19
20	HOPI MOENCOPI	ARKOT-8102	18
20	HOPI MOENCOPI	CAHUGLBBCS-1-88	18
20	HOPI MOENCOPI	COKER-201	18
20	HOPI MOENCOPI	SPNXCHGLBH-1-94	19
37	SPNXCHGLBH-1-94	ARKOT8102	18
37	SPNXCHGLBH-1-94	CABD3CABCH-1-89	20
37	SPNXCHGLBH-1-94	GSA74	19
37	SPNXCHGLBH-1-94	TAMCOT SP-23	20
39	TAMCOT SP-23	ARKOT-8102	20
39	TAMCOT SP-23	CABD3CABCH-1-89	22
39	TAMCOT SP-23	GSA74	21
39	TAMCOT SP-23	SPNXCHGLBH-1-94	20
Total			587

Table S4. List of the F_2 populations (received from Costa Rica) sown at the Pee Dee REC.

Cross ID	Cross Combination	Seeds Sown	Seeds Germinated
8x5	CABD3CABCH-1-89 × ARKOT-8102	230	102
8x39	CABD3CABCH-1-89 × TAMCOT SP-23	235	107
37x39	SPNXCHGLBH-1-94 × TAMCOT SP-23	220	103
17x5	GSA 74 × ARKOT-8102	243	106
17x39	GSA 74 × TAMCOT SP-23	246	93

Table S5. List of the F_1 genotypes sown at the Pee Dee REC.

Cross ID	Cross Combination	Seeds Sown	Seeds Germinated
5x8	ARKOT 8102 × CABD3CABCH-1-89	122	95
5x17	ARKOT 8102 × GSA 74	98	69
5x37	ARKOT 8102 × SPNXCHGLBH-1-94	104	76
5x39	ARKOT 8102 × TAMCOT SP-23	50	23
8x17	CABD3CABCH-1-89 × GSA 74	50	30
8x37	CABD3CABCH-1-89 × SPNXCHGLBH-1-94	104	72
17x8	GSA 74 × CABD3CABCH-1-89	122	73
17x37	GSA 74 × SPNXCHGLBH-1-94	50	0
37x5	SPNXCHGLBH-1-94 × ARKOT 8102	86	37
37x8	SPNXCHGLBH-1-94 × CABD3CABCH-1-89	128	79
37x17	SPNXCHGLBH-1-94 × GSA 74	50	41
39x5	TAMCOT SP-23 × ARKOT 8102	50	42
39x8	TAMCOT SP-23 × CABD3CABCH-1-89	50	39
39x17	TAMCOT SP-23 × GSA 74	50	27
39x37	TAMCOT SP-23 × SPNXCHGLBH-1-94	50	23

Table S6. List of F_1 lines sent for generation advancement at Costa Rica in the winter nursery 2019-2020.

Plant ID	Tab#	Genotype Combinations (genetic crosses)
SR19-61 (0-4)	484	CABD3CABCH-1-89 × CAHUGLBBCS-1-88
SR19-62 (0-4)	460	CAHUGLBBCS-1-88 × SPNXCHGLBH-1-94
SR19-63 (0-4)	1618	HOPI MOENCOPI × SPNXCHGLBH-1-94
SR19-64 (0-4)	1064	CAHUGLBBCS-1-88 × ARKOT-8102
SR19-65 (0-4)	1865	HOPI MOENCOPI × CAHUGLBBCS-1-88
SR19-66 (0-4)	652	CAHUGLBBCS-1-88 × COKER-201
SR19-67 (0-4)	1301	CABD3CABCH-1-89 × COKER-201
SR19-68 (0-4)	1734	HOPI MOENCOPI × COKER-201
SR19-69 (0-4)	118	CABD3CABCH-1-89 × HOPI MOENCOPI
SR19-70 (0-4)	422	HOPI MOENCOPI × ARKOT-8102

Table S7. The allele-specific genotyping of the F_2 population (17x39) with i09222Gh, i00443Gh, i13158Gh, and i13851Gh molecular markers.

Sample ID	18srRNA	i09222Gh	i00443Gh	i13158Gh	i13851Gh
1	+	CT	CC	AA	AA
2	+	*	*	*	AA
3	+	TT	CC	*	*
4	+	CT	*	*	*
5	+	*	*	*	*
6	+	TT	*	*	*
7	+	*	*	*	*
8	+	*	*	*	*
9	+	*	*	*	*
10	+	CT	TT	AG	AG
11	+	CT	*	*	AG
12	+	*	*	*	*
13	+	*	*	*	*
14	+	*	*	*	*
15	+	TT	CC	AG	*
16	+	CT	TC	AG	*
17	+	CC	*	*	*
18	+	TT	*	*	*
19	+	CT	CC	AG	AG
20	+	CC	CC	GG	*
21	+	CC	*	*	*
22	+	TT	*	*	*
23	+	TT	*	AG	*
24	+	*	*	AG	*
25	+	*	*	*	*
26	+	*	*	*	*
27	+	CC	TC	AG	GG
28	+	TT	TC	AG	GG
29	+	TT	*	*	GG
30	+	*	*	*	*
31	+	*	*	*	*
32	+	CT	CC	AG	*
33	+	*	*	*	GG
34	+	CT	CC	AG	AG

35	+	CT	CC	*	AG
36	+	CT	*	AA	AG
37	+	CC	*	AA	AA
38	+	*	*	*	AG
39	+	TT	*	AA	AA
40	+	*	*	AA	*
41	+	*	*	AA	GG
42	+	CC	*	*	*
43	+	*	*	*	*
44	+	*	CC	AG	*
45	+	*	TT	*	*
46	+	*	TC	AG	GG
47	+	*	TT	AA	GG
48	+	*	*	*	AG
49	+	TT	TC	AG	*
50	+	*	*	*	*
51	+	*	*	*	*
52	+	*	*	*	*
53	+	*	*	*	*
54	+	*	*	*	*
55	+	*	*	*	*
56	+	*	*	*	*
57	+	*	*	*	*
58	+	*	*	*	*
59	+	CC	*	AA	AA
60	+	CC	*	*	*
61	+	CT	TC	AA	AG
62	+	CC	*	*	*
63	+	CT	CC	AA	GG
64	+	*	*	*	*
65	+	*	*	*	*
66	+	*	*	*	*
67	+	*	TC	AG	AG
68	+	*	*	*	*
69	+	*	*	*	*
70	+	*	*	*	*
71	+	*	*	*	*
72	+	CT	TT	GG	AA
73	+	CC	CC	AG	AA

74	+	CT	TC	AG	AA
75	+	*	*	AA	GG
76	+	CT	CC	AG	GG
77	+	*	*	*	*
78	+	*	*	*	*
79	+	CT	TC	AG	GG
80	+	*	*	*	*
81	+	*	*	*	AG
82	+	*	CC	*	*
83	+	*	*	*	*
84	+	*	*	*	*
85	+	CT	TC	AG	AG
86	+	CT	TC	AG	AG
87	+	*	*	*	*
88	+	TT	*	*	AG
89	+	CT	*	GG	AG
90	+	CC	*	GG	AG
91	+	*	*	*	*

Note 1: * the plants did not produce any data with these molecular markers.
Note 2: + shows positive results with 18SrRNA.

Table S8. List of F_2 plants selected for propagation in Costa Rica in 2020.

Plant Id	Selections for F_3
**CABD3CABCH-1-89 (8) x CAHUGLBBCS-1-88 (9)**	
P3	SNR-8x9-301
P6	SNR-8x9-302
P28	SNR-8x9-303
P30	SNR-8x9-304
P38	SNR-8x9-305
**CAHUGLBBCS-1-88 (9) x ARKOT 8102 (5)**	
P8	SNR-9x5-401
P16	SNR-9x5-402
P19	SNR-9x5-403
P22	SNR-9x5-404
P24	SNR-9x5-405
P33	SNR-9x5-407
**HOPI MOENCOPI (20) x ARKOT 8102 (5)**	
P2	SNR-20x5-501
P21	SNR-20x5-502
P22	SNR-20x5-503
P23	SNR-20x5-504
P29	SNR-20x5-505
**TAMCOT SP-23 (39) x ARKOT 8102 (5)**	
P5	SNR-39x5-601
P14	SNR-39x5-602
P17	SNR-39x5-603
P19	SNR-39x5-604
**SPNXCHGLBH-1-94 (37) x CABD3CABCH-1-89 (8)**	
P7	SNR-37x8-201
P19	SNR-37x8-202
**CABD3CABCH-1-89 (8) x SPNXCHGLBH-1-94 (37)**	
P1	SNR-8x37-801
P2	SNR-8x37-802
**HOPI MOENCOPI (20) x SPNXCHGLBH-1-94 (37)**	
P9	SNR-20x37-901
P24	SNR-20x37-902
P33	SNR-20x37-903
P36	SNR-20x37-904

Table S9. List of F_3 plants selected for propagation in Costa Rica in 2020.

Plant Id	Selections for F_4	Remark
**SPNXCHGLBH-1-94 (37) x TAMCOT SP-23 (39)**		
P1-2	SNR-37/39-5001-2	LE
P1-3	SNR-37/39-5001-3	LE
P1-4	SNR-37/39-5001-4	LE
P1-5	SNR-37/39-5001-5	LE
P1-6	SNR-37/39-5001-6	LE
P6-11	SNR-37/39-706-11	HE
P3-2	SNR-37/39-703-2	HE
P4-4	SNR-37/39-704-4	HE
**GSA 74 (17) x ARKOT 8102 (5)**		
P1-2	SNR-17/5-3001-2	LE
P1-7	SNR-17/5-3001-7	LE
P8-1	SNR-17/5-108-1	HE
P8-2	SNR-17/5-108-2	HE
P01-2	SNR-17/5-101-2	HE
**CABD3CABCH-1-89 (8) x TAMCOT SP-23 (39)**		
P5-1	SNR-8/39-2005-1	LE
P5-2	SNR-8/39-2005-2	LE
P5-5	SNR-8/39-2005-5	LE
P5-6	SNR-8/39-2005-6	LE
P6-7	SNR-8/39-006-7	HE
**CABD3CABCH-1-89 (8) x ARKOT 8102 (5)**		
P4-1	SNR-8/5-4-1	HE
P5-1	SNR-8/5-5-1	HE
P5-6	SNR-8/5-5-6	HE
P5-8	SNR-8/5-5-8	HE
P6-9	SNR-8/5-6-9	HE
P9-3	SNR-8/5-9-3	HE
P10-4	SNR-8/5-10-4	HE

Note: Plants from two extremes of the population distribution of the phenotypic traits (flower number, flowering time, timing of first boll opening, and plant height) recorded in 2019. HE = plants showing higher phenotypic expression and LE = lower phenotypic expression for the studied phenotypic traits.

Table S10. List of the populations sown at PDREC research fields in 2021.

Lines	Seeds Germinated	Seeds Sown	Germination Percentage
**F_4-Costa Rica**			
17x5-101-2	49	100	49
8x5-10-4	41	100	41
**F_3-Costa Rica**			
9x5-406	61	100	61
9x5-402	0	100	0
20x37-904	52	100	52
20x37-901	22	100	22
8x9-304	15	100	15
39x5-604	50	100	50
20x5-504	49	100	49
**F_3-PDREC-2020**			
37x39-706-11	0	100	0
37x39-704-4	0	100	0
37x39-703-2	12	100	12
37x39-5001-2	16	100	16
37x39-5001-3	11	100	11
37x39-5001-4	12	100	12
37x39-5001-5	1	100	1
37x39-5001-6	24	100	24
8x5-4-1	30	100	30
8x5-5-1	0	100	0
8x5-5-6	1	100	1
8x5-5-8	50	100	50
8x5-6-9	19	100	19
8x5-9-3	29	100	29
8x39-006-7	34	100	34
8x39-2005-1	26	100	26
8x39-2005-2	31	100	31
8x39-2005-5	20	100	20
8x39-2005-6	0	100	0
17x5-3001-2	6	100	6
17x5-3001-7	18	100	18
17x5-108-1	7	100	7
17x5-108-2	6	100	6
**F_2-PDREC-2020**			
20x5-501	20	100	20
20x5-502	0	100	0
20x5-503	0	100	0
20x5-505	14	100	14

20x37-902	42	100	42
8x37-801	0	100	0
8x37-802	9	100	9
8x9-301	28	100	28
8x9-302	0	100	0
8x9-303	0	100	0
8x9-305	9	100	9
9x5-401	49	100	49
9x5-403	54	100	54
9x5-405	23	100	23
39x5-601	35	100	35
39x5-602	26	100	26
39x5-603	18	100	18
20x37-903	0	100	0

Table S11. List of F_4 plants selected for propagation in Costa Rica.

**Plant Id**	**Selections for F_4**
**CAHUGLBBCS-1-88 (9) x ARKOT 8102 (5)**	
P1	SNR-9x5-406-C1
P41	SNR-9x5-406-C2
**HOPI MOENCOPI (20) x SPNXCHGLBH-1-94 (37)**	
P46	SNR-20x37-904-D1
P52	SNR-20x37-904-D2
P53	SNR-20x37-904-D3
**HOPI MOENCOPI (20) x SPNXCHGLBH-1-94 (37)**	
P8	SNR-20x37-901-K1
P14	SNR-20x37-901-K2
**CABD3CABCH-1-89 (8) x CAHUGLBBCS-1-88 (9)**	
P15	SNR-8x9-304-E1
P16	SNR-8x9-304-E2
P17	SNR-8x9-304-E3
**TAMCOT SP-23 (39) x ARKOT 8102 (5)**	
P17	SNR-39x5-604-H1
P30	SNR-39x5-604-H2
**HOPI MOENCOPI (20) x ARKOT 8102 (5)**	
P23	SNR-20x5-504-J1
P36	SNR-20x5-504-J2
P42	SNR-20x5-504-J3
P43	SNR-20x5-504-J4
P46	SNR-20x5-504-J5

Table S12. List of F_5 plants selected for propagation in Costa Rica.

Plant Id	Selections for F_5
**GSA 74 (17) x ARKOT 8102 (5)**	
P21	SNR-17x5-101-2-A1
P32	SNR-17x5-101-2-A2
P34	SNR-17x5-101-2-A3
P35	SNR-17x5-101-2-A4
P33	SNR-17x5-101-2-A5
**CABD3CABCH-1-89 (8) x ARKOT 8102 (5)**	
P27	SNR-8x5-10-4-B1
P31	SNR-8x5-10-4-B2
P33	SNR-8x5-10-4-B3
P39	SNR-8x5-10-4-B4
P41	SNR-8x5-10-4-B5

Table S13. The list of selected genotypes sent for generation advancement (F_9/F_8) to Costa Rica in 2022.

Genotype	Plant Number					Block Number
20x37-K1	6	7*	9	8	5	2
20x37-K2	6	3*	10	5	8	2
20x37-D1	6	4	5*	9	7	1
20x5-J3	4	6*	3	9		2
20x5-J2	7	5*	10	2		3
39x5-H1	9	6*	1			3
8x9-E3	8	3*	4			1
8x9-E2	8	1	2*	4		3
9x5-C1	5	9*	10	1		1
8x5-B1	9	6*	5	8		2
8x5-B4	4	2	10*	9		1
8x5-B3	10*	9	6	3		2
17x5-A5	7*	8	3	5		3
17x5-A2	4*	5	1	8		3
17x5-A1	5	4*	6	9		2

*Selected plants sent to Costa Rica for an increase.
